# Random Mutagenesis of the *Aspergillus oryzae* Genome Results in Fungal Antibacterial Activity

**DOI:** 10.1155/2013/901697

**Published:** 2013-07-28

**Authors:** Cory A. Leonard, Stacy D. Brown, J. Russell Hayman

**Affiliations:** ^1^Department of Biomedical Sciences, James H. Quillen College of Medicine, East Tennessee State University, P.O. Box 70577, Johnson City, TN 37614, USA; ^2^Department of Pharmaceutical Sciences, Bill Gatton College of Pharmacy, East Tennessee State University, P.O. Box 70436, Johnson City, TN 37614, USA

## Abstract

Multidrug-resistant bacteria cause severe infections in hospitals and communities. Development of new drugs to combat resistant microorganisms is needed. Natural products of microbial origin are the source of most currently available antibiotics. We hypothesized that random mutagenesis of *Aspergillus oryzae* would result in secretion of antibacterial compounds. To address this hypothesis, we developed a screen to identify individual *A. oryzae* mutants that inhibit the growth of Methicillin-resistant *Staphylococcus aureus* (MRSA) *in vitro*. To randomly generate *A. oryzae* mutant strains, spores were treated with ethyl methanesulfonate (EMS). Over 3000 EMS-treated *A. oryzae* cultures were tested in the screen, and one isolate, CAL220, exhibited altered morphology and antibacterial activity. Culture supernatant from this isolate showed antibacterial activity against Methicillin-sensitive *Staphylococcus aureus*, MRSA, and *Pseudomonas aeruginosa*, but not *Klebsiella pneumonia* or *Proteus vulgaris*. The results of this study support our hypothesis and suggest that the screen used is sufficient and appropriate to detect secreted antibacterial fungal compounds resulting from mutagenesis of *A. oryzae*. Because the genome of *A. oryzae* has been sequenced and systems are available for genetic transformation of this organism, targeted as well as random mutations may be introduced to facilitate the discovery of novel antibacterial compounds using this system.

## 1. Introduction

Antibiotic resistant microorganisms became a medical concern shortly after antibiotics became readily available in the 1940s. In fact, resistance has typically been identified within four years of Food and Drug Administration approval of antibacterial agents [1]. Currently, multidrug-resistant bacteria such as MRSA, coagulase negative Staphylococci, and Enterococci cause severe infections in both hospital and community settings [2]. Development of new drugs to combat the increasing host of drug resistant microorganisms is essential if we are to avoid the emergence of pathogens for which there exist no effective antimicrobial therapies.

Most classes of antibiotics were developed from natural products produced by fungi or filamentous bacteria. Significantly, the majority of antibiotics still commonly used today are natural product compounds, or their derivatives, discovered during the “golden era” of antibiotic discovery from the 1940s through the 1960s [3]. More recent efforts to generate new antibiotics based on high-throughput, target-focused screening of large libraries of synthetic compounds have largely failed to produce new antibiotics [3, 4]. Generation of novel antibiotic compounds may be possible by revealing previously untapped chemical diversity in known natural products. For example, recent studies have shown that mutations in microbes can activate silent gene clusters, regulating enzyme activity, and resulting in the production of uncharacterized compounds, some of which exhibit antimicrobial activity [5]. 

*Aspergillus oryzae* is a domesticated filamentous fungus used in the food fermentation industry. Although *A. oryzae* is known to produce antibacterial compounds, such as kojic acid and aspirochlorine, the fungus does not produce significant levels of these compounds under standard food industry or laboratory culture conditions. Recent work, however, utilizing manipulation of culture conditions, suggests that *A. oryzae*, in common with the closely related *Aspergillus flavus*, has the potential to produce a broader range of secondary metabolites than previously thought possible [6]. Random mutagenesis might facilitate the production of antibacterial compounds by altering expression of polyketide synthase or nonribosomal peptide synthetase pathways, the main pathways involved in fungal secondary metabolism [7]. Additionally, such mutagenesis could modulate regulation of protein expression and production of antimicrobial peptides. Filamentous fungi, including *Aspergillus giganteus* and *Aspergillus clavatus*, are known to produce antimicrobial peptides with demonstrated antifungal or antibacterial activity [8]. It is our hypothesis that random mutagenesis of *A. oryzae* may result in strains secreting novel antibacterial compounds, or increased levels of known antibacterial compounds to detectable levels. To address this hypothesis, a biological screening assay was employed to identify individual *A. oryzae* mutants that secrete factors inhibiting the growth of Methicillin-resistant *Staphylococcus aureus* (MRSA) *in vitro*.

## 2. Materials and Methods

### 2.1. Bacterial and Fungal Strains

Methicillin-sensitive *Staphylococcus aureus* (ATCC 25922) and *Pseudomonas aeruginosa* (ATCC 27853), as well as Methicillin-resistant *Staphylococcus aureus* (MRSA), *Klebsiella pneumonia*, and *Proteus vulgaris* (clinical isolates), were generously provided by Dr. Donald A. Ferguson, Jr. Ph.D., Director of the Clinical Microbiology Laboratory, Quillen College of Medicine, East Tennessee State University. *Aspergillus oryzae* (FGSC#A815) was obtained from the Fungal Genetic Stock Center, University of Missouri-Kansas City. Bacterial strains were maintained on blood agar plates at 37°C and *A. oryzae* was maintained on Sabourad dextrose agar (SAB) plates at 30°C until screening *A. oryzae* culture supernatants for antibacterial activity (see below).

### 2.2. Ethyl Methanesulfonate (EMS) Mutagenesis of *Aspergillus oryzae*

*A. oryzae* spores (Fungal Genetics Stock Center), in sterile water, were treated with zero (mock), 50, 100, 200, 400, 800, 1600, 3200, or 6400 *μ*g mL^−1^ EMS overnight at 37°C. Washed and diluted spores were then plated onto SAB plates and incubated at 37°C for 48 hours. The percentage of viable EMS-treated spores compared to mock treated spores was determined by counting fungal colonies per plate. 

### 2.3. Screening *A. oryzae* Culture Supernatants for Antibacterial Activity

#### 2.3.1. Preparation of *A. oryzae* Culture Supernatants

*A. oryzae* nontreated, mock-treated, and 1600 *μ*g mL^−1^ EMS-treated spores were separately cultured in flat-bottom 96 well plates in Luria-Bertani (LB) liquid media at a volume of 200 *μ*L per well at an estimated spore density of one or less viable spores per well. The plates were incubated at 37°C for four days with no shaking. 

#### 2.3.2. Screening Assay of Supernatant Activity against MRSA

After four days of fungal culture, 100 *μ*L of *A. oryzae* culture supernatant was transferred from each well of the 96 well culture plates to wells in U-bottom 96 well plates containing 100 *μ*L fresh LB media (to provide nutrients that may have been depleted by fungal growth) and 10 *μ*L of 0.005 OD600 MRSA suspended in sterile phosphate buffered saline (PBS). Positive control wells for MRSA growth consisted of 200 *μ*L LB media with no *A. oryzae* culture supernatant plus the described MRSA inoculum. Negative control wells consisted of 200 *μ*L LB media with no bacterial inoculum. The plates were incubated overnight at 37°C with no shaking and MRSA growth in the presence of 3080 EMS-treated fungal culture supernatants was visually evaluated. Images were captured using an Epson Perfection 3200 Photo scanner. The remaining fungal culture supernatant volumes and associated mycelia mats were stored at 4°C, tightly sealed in the original 96 well fungal culture plates. 

#### 2.3.3. Dilution/Isolation of EMS-Treated Putative Antibacterial *A. oryzae*

Fungal cultures that yielded supernatant samples with visible antibacterial activity, as determined by reduced bacterial growth in the screening assay, were diluted and isolated. The corresponding fungal mats were retrieved from 4°C storage and subcultured onto SAB plates at 37°C. When fungal colony growth was just visible, sterile wooden applicator sticks were used to transfer mycelia growth to gridded SAB plates and cultures at 37°C. Colonies were observed for morphological differences and retested by repeated fungal culture and screening assay.

### 2.4. Characterization of Fungal Colony Size and Onset of Spore Production

The single isolated antibacterial *A. oryzae* isolate and the parental *A. oryzae* strain were inoculated onto SAB plates, 300 spores in 10 *μ*L sterile water at the plate center, and cultured at 37°C for two days, then moved to room temperature for further culture. A Westover Scientific Stereo Microscope and Sony HQX Digital Still Camera were used to observe colony size and spore formation at two through seven days after inoculation. Colony diameter sizes were quantitated in cm.

### 2.5. Characterization of Spore Production

The antibacterial *A. oryzae* isolate and the parental *A. oryzae* strain were inoculated onto SAB plates, 3000 spores in 100 *μ*L sterile water spread to form lawns of fungal growth, and cultured at 37°C for two days then moved to room temperature for further culture. At eight days after inoculation, fungal growth and spore appearance had not visibly changed for several days for either isolate and spores were harvested and counted. Briefly, 1.5 cm diameter agar plugs comprising confluent mycelia growth and visibly densest spore growth were excised from the parental strain and antibacterial isolate *A. oryzae* plates and placed into tightly capped 50 mL tubes with 10 mL of sterile water. The tubes were shaken vigorously to dislodge the fungal spores into the water (5 × 30 seconds) and the agar plugs were discarded from the tubes. Undiluted samples were counted using a hemacytometer and an Olympus BH-2 microscope at 40x magnification. Three counts were performed per sample to determine mean spore concentration, reported as spores mL^−1^.

### 2.6. Effect of Antibacterial *A. oryzae* Supernatant on Various Bacteria

Parental *A. oryzae* spores and antibacterial *A. oryzae* isolate spores were cultured in LB liquid media in 6 well flat-bottom plates at a spore concentration of 5 × 10^3^ spores mL^−1^. The cultures were incubated at 37°C for seven days with no shaking and filtered (0.22 *μ*m). 100 *μ*L aliquots of the filtered culture supernatants were transferred to wells in U-bottom 96 well plates containing 100 *μ*L fresh LB media and 10 *μ*L of a dilute bacterial suspension (0.005 OD600 bacteria suspended in sterile PBS). Positive control wells for bacterial growth consisted of 200 *μ*L LB media with no culture supernatant plus the described bacterial inoculum. Negative control wells consisted of 200 *μ*L LB media with no bacterial inoculum. The plates were incubated overnight at 37°C with no shaking and bacterial growth in the presence of fungal culture supernatant was visually evaluated. The following bacteria were evaluated as described: Methicillin-sensitive *Staphylococcus aureus*, Methicillin-resistant *Staphylococcus aureus*, *Pseudomonas aeruginosa*, *Klebsiella pneumonia*, and *Proteus vulgaris*.

### 2.7. Liquid Chromatography-Mass Spectroscopy (LC-MS)

The (0.22 *μ*m) filtered parental strain and antibacterial isolate *A. oryzae* culture supernatants were analyzed by LC-MS/MS. Fresh 0.22 *μ*m filtered LB liquid media served as a control. Chromatography was performed on a Shimadzu liquid chromatography system using a Thermo Aquasil C-18 column (150 × 4.6 mm, 5 *μ*m particle size) at a flow rate of 0.25 mL minute^−1^. The system was coupled to the Shimadzu ion trap-time of flight (IT-TOF) mass spectrometer with an electrospray (ESI) source and consisted of two LC-20AD pumps with UFLC-XR upgrade, SIL-20ACHT autosampler, CTO-20A column oven, DGU-20A_3_ degasser, and CBM-20A communications module. Data was analyzed with LCMSSolution and Profiler software (Shimadzu). Aliquots of the LB control and both fungal culture supernatants were eluted with a mobile phase gradient of 10% acetonitrile, 0.1% formic acid (90% H_2_O) at one minute to 90% acetonitrile, and 0.1% formic acid (10% H_2_O) at 20 minutes.

## 3. Results and Discussion

**Figure 1:**
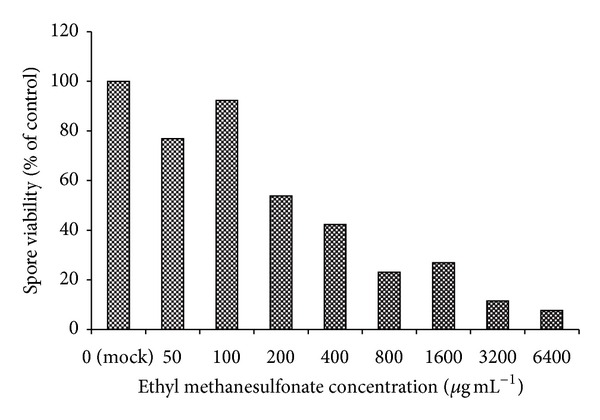
Ethyl methanesulfonate mutagenesis of *Aspergillus oryzae*. *Aspergillus oryzae* spore viability was reduced in a dose-dependent manner by ethyl methanesulfonate (EMS) treatment. Spore treatment with 1600 *μ*g mL^−1^ EMS was used to generate the *A. oryzae* mutants screened for secretion of antibacterial compounds.

In the present study we designed a screening assay to evaluate antibacterial activity of *A. oryzae* culture supernatants from liquid fungal culture. Ethyl Methanesulfonate (EMS) at a concentration range from 50 to 6400 *μ*g mL^−1^ was employed to produce mutations in the *A. oryzae* genomic DNA. Spore treatment with EMS concentration of 1600 *μ*g mL^−1^ resulted in approximately 25% of control viability (see Figure 1) and resulted in visibly smaller fungal colony sizes on SAB agar plates compared to mock treatment or treatment with lower EMS concentrations. Previously, 25% spore viability of *Aspergillus niger* was the minimum spore lethality induced by EMS sufficient to produce detectable auxotrophic mutants amongst treated spores [9]. Although EMS treatment resulting in higher levels of spore lethality would result in higher levels of mutation frequency, posttreatment spore viability of approximately 25% was selected to minimize the number of mutations per resulting fungal strain. Spore treatment with EMS concentration of 1600 *μ*g mL^−1^ was subsequently used to generate a pool of potential *A. oryzae* mutants, which were screened for the secretion of antibacterial compounds. 

**Figure 2:**
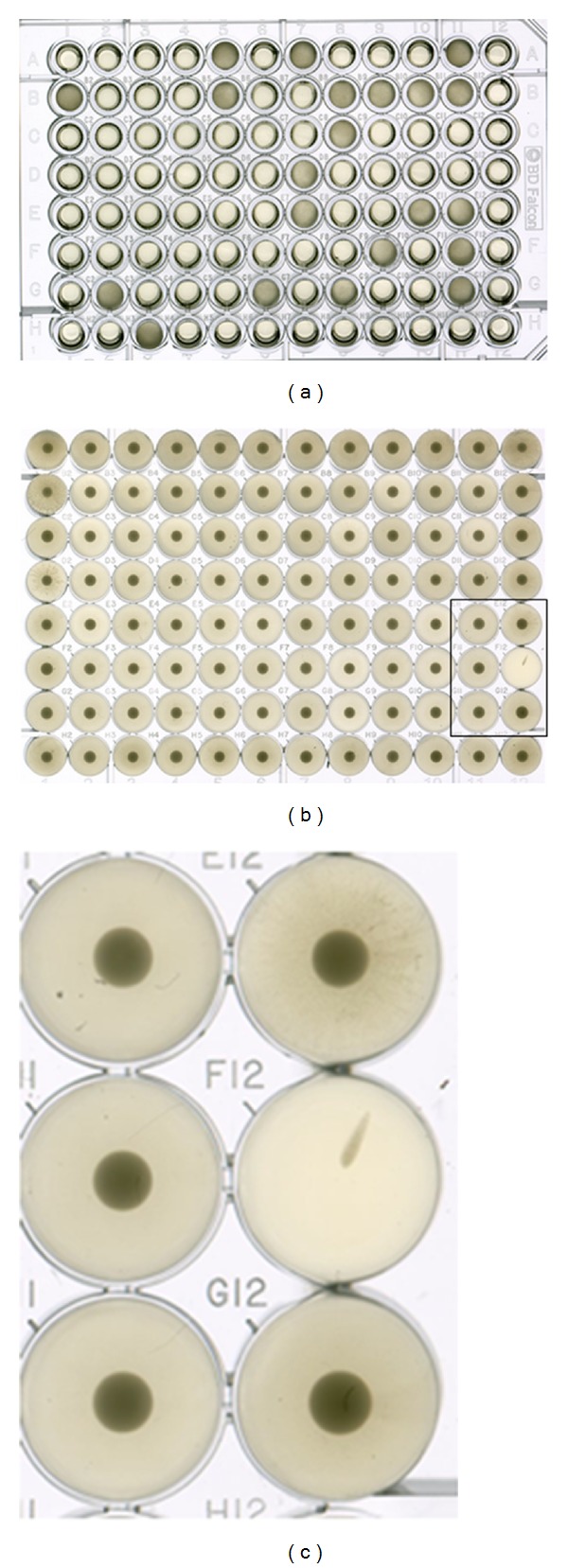
Screening *A. oryzae* culture supernatants for antibacterial activity. Fungal culture in 96 well plates allows visible confirmation of fungal growth. As seen in (a), wells with fungal growth are dark, while wells with no fungal growth contain only culture media and are comparatively clear. Examination of U-bottom 96 well plate bacterial cultures subjected to fungal culture supernatants allows visual evaluation of reduction in MRSA bacterial growth (b). (c) a magnified area (boxed) of image in (b) clearly showing a single well with extremely reduced bacterial growth indicating antibacterial activity of fungal culture supernatant.

The screening method used in this study allowed small-volume (200 *μ*L) liquid culture of putative mutant *A. oryzae* spores, visible confirmation of fungal growth in 96 well fungal culture plates (see Figure 2(a)), and visual examination of U-bottom 96 well plate bacterial cultures subjected to fungal culture supernatants for reduction in MRSA bacterial growth (see Figures 2(b) and 2(c)). While manipulation of fungal culture media and genetic mutation can both be utilized to alter expression of fungal metabolites [5, 6], mutation reduces the need for further manipulation of fungal supernatants containing possible secreted antibacterial compounds prior to screening for activity. In our study, because the EMS-treated spores were cultured in standard bacterial culture media (LB media), antibacterial activity of fungal culture supernatants could be assayed directly without extraction of active compounds and reconstitution in bacteria compatible media, which would require larger fungal culture volumes. Mutant fungal strains identified by this method may be later subjected to culture in various medias or conditions to determine if such manipulations can contribute to increased metabolic diversity and/or metabolite yield.

Over 3000 EMS-treated, putative mutant *A. oryzae* cultures were tested in the described screening assay for antibacterial activity. A single EMS-treated *A. oryzae* isolate, named CAL220, exhibited antibacterial activity in the screening assay as indicated by complete lack of visible MRSA growth in the assay. Subculture of assay well contents to LB agar plates showed no bacterial growth and confirmed that the fungal culture supernatant killed the MRSA bacteria. Repeated assay of filter sterilized culture supernatant from the antibacterial *A. oryzae* isolate showed antibacterial activity against *Staphylococcus aureus* (Methicillin-sensitive), MRSA, and *Pseudomonas aeruginosa*, but not *Klebsiella pneumonia* or *Proteus vulgaris*.

The compound(s) in the antibacterial *A. oryzae* culture supernatant responsible for the observed antibacterial activity have thus far not been isolated, so minimum inhibitory concentrations cannot be determined at this time. However, dose-dependent effect of the antibacterial culture supernatant was evaluated by employing serial dilutions in LB media of the supernatant in the screening assay. Activity against *Staphylococcus aureus*, MRSA, and *Pseudomonas aeruginosa* was confirmed in 1 : 2 diluted and 1 : 4 diluted antibacterial culture supernatant, while further dilutions of the supernatant (1 : 8, 1 : 16, 1 : 32) resulted in lack of complete or detectable antibacterial activity indicating that the antibacterial compound is present in low concentration in the active supernatant (data not shown). 

Preliminary liquid chromatography-tandem mass spectrometric (LC-MS/MS) analysis indicates that the antibacterial fungal culture supernatant contains a single small molecule species of *m*/*z* 602 with a fragment ion of *m*/*z* 245 not found in the parental fungal culture supernatant or in the culture media alone (data not shown). While the identity of this compound has not yet been elucidated, the *m*/*z* value indicates a possible molecular weight of 601 Da, which would differentiate it from lower molecular weight compounds such as kojic acid, aspirochlorine, aflatoxin, asperfuran, cyclopiazonic acid, 3-nitropropionic acid, maltoryzine, sporogen, aflatrem, 13-desoxypaxilline, miyakamides, parasiticolide, and aspergillomarasmine, compounds which have been previously isolated from *A. oryzae* and/or the 99.5% genetically homologous *A. flavus* [6]. Heat sterilizing the active supernatant (100°C 5–20 minutes) did not diminish antibacterial activity, while lyophilization and reconstitution in smaller volumes in an attempt to concentrate the active compound did reduce activity. This activity reduction is likely due to increased NaCl_2_ concentration as increasing NaCl_2_ concentration to three fold in excess of LB concentration (10 g L^−1^) in nonlyophilized samples also reduced activity. 

**Figure 3:**
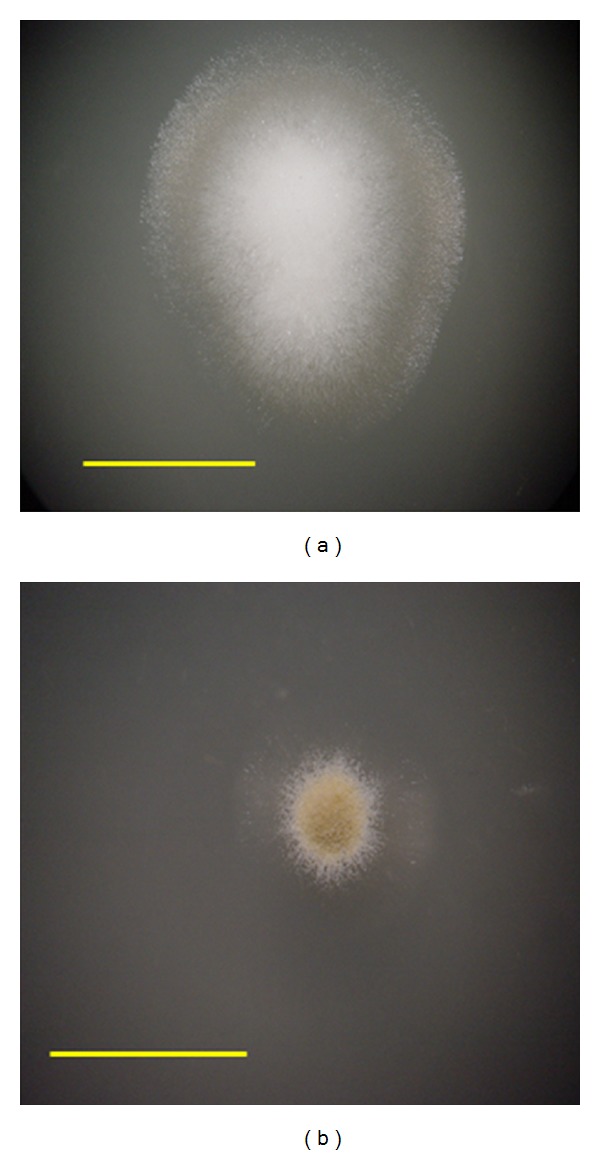
Characterization of mutant *Aspergillus oryzae* colony size and onset of spore production. The antibacterial *A. oryzae* isolate (b) exhibited altered morphology consisting of smaller colony size, reduced mycelia formation, and earlier and heavier visible spore production compared to parental *A. oryzae* (a). Colonies are shown at two days after inoculation on SAB plates. Without magnification, spores are visible as dark coloration (b), while mycelia are bright white (a). Scale bars = 0.75 cm.

The antibacterial *A. oryzae* isolate exhibited altered morphology consisting of smaller colony size, reduced mycelia formation, and earlier and heavier visible spore production (see Figure 3). At all times from one to seven days after inoculation, the parental colony exhibited dense, fluffy mycelia growth, and little spore production, while the antibacterial isolate colony exhibited minimal mycelia growth with heavy spore production. Spore production quantitated from equivalent fungal SAB cultures showed that parental strain *A. oryzae* yielded less than 1 × 10^4^ spores mL^−1^, while antibacterial isolate *A. oryzae* yielded approximately 1.5 × 10^6^ spores mL^−1^ when using an equal volume of eluent, more than 100 times as many spores as the parental strain.

Future work in our laboratory will include fractionation and isolation of the antibacterial compound(s) from the mutant *A. oryzae* isolate generated in this study. The results of this study support our hypothesis that random mutations introduced into the *A. oryzae* genome can result in fungal secretion of antibacterial factors and suggest that the screen used is sufficient and appropriate to detect secreted antibacterial fungal compounds resulting from mutagenesis. The genome of *A. oryzae* has been sequenced, and systems are available for genetic transformation of this organism. Thus targeted as well as random mutations may be introduced to facilitate the discovery of novel antibacterial compounds using the screening system reported here.
